# A robust and readily implementable method for the meta‐analysis of response ratios with and without missing standard deviations

**DOI:** 10.1111/ele.14144

**Published:** 2022-12-26

**Authors:** Shinichi Nakagawa, Daniel W. A. Noble, Malgorzata Lagisz, Rebecca Spake, Wolfgang Viechtbauer, Alistair M. Senior

**Affiliations:** ^1^ Evolution & Ecology Research Centre and School of Biological, Earth and Environmental Sciences University of New South Wales Sydney New South Wales Australia; ^2^ Division of Ecology and Evolution, Research School of Biology The Australian National University Canberra Australian Capital Territory Australia; ^3^ School of Biological Sciences University of Reading Reading UK; ^4^ Faculty of Health, Medicine, and Life Sciences, Department of Psychiatry and Neuropsychology, School for Mental Health and Neuroscience Maastricht University Maastricht The Netherlands; ^5^ Charles Perkins Centre, School of Life and Environmental Sciences and Sydney Centre for Precision Data Science University of Sydney New South Wales Camperdown Australia

**Keywords:** meta‐regression, missing data, multiple imputation, research synthesis, robust variance estimation

## Abstract

The log response ratio, lnRR, is the most frequently used effect size statistic for meta‐analysis in ecology. However, often missing standard deviations (SDs) prevent estimation of the sampling variance of lnRR. We propose new methods to deal with missing SDs via a weighted average coefficient of variation (CV) estimated from studies in the dataset that do report SDs. Across a suite of simulated conditions, we find that using the average CV to estimate sampling variances for all observations, regardless of missingness, performs with minimal bias. Surprisingly, even with missing SDs, this simple method outperforms the conventional approach (basing each effect size on its individual study‐specific CV) with complete data. This is because the conventional method ultimately yields less precise estimates of the sampling variances than using the pooled CV from multiple studies. Our approach is broadly applicable and can be implemented in all meta‐analyses of lnRR, regardless of ‘missingness’.

## Graphical abstract

We propose four new methods to deal with missing standard deviations (SDs) in meta‐analyses of log response ratio (lnRR). Our simulation shows that all the methods perform well and, rather surprisingly, two of our methods with missing data perform better than traditional methods without missing data. All future meta‐analyses of lnRR can take advantage of our methods.

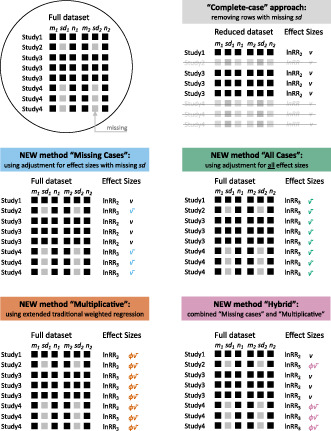

## INTRODUCTION

Meta‐analyses are frequently used to quantitatively synthesise the outcomes of ecological studies and explain inconsistencies among findings (Gurevitch et al., 2018). Meta‐analyses often compare the means of two groups, and the most widely used effect sizes for this are the standardised mean difference, SMD (i.e. Cohen's *d* and Hedges' *g*), and the natural logarithm of the response ratio, lnRR (Hedges et al., 1999; Koricheva & Gurevitch, 2014; Nakagawa & Santos, 2012). Both the SMD and lnRR require the standard deviations (SDs) of the two groups to estimate the effect size's precision (i.e. sampling variance). However, many empirical papers do not report SDs or statistics from which SDs can be calculated (e.g. standard errors). A recent review found incomplete reporting of SDs is pervasive and threatens the validity of meta‐analytic evidence. Of 505 ecological meta‐analytic studies, nearly 70% of the datasets included studies with missing SDs (Kambach et al., 2020). The same review also showed that many meta‐analysts exclude studies with missing SDs, also known as a ‘complete‐case’ analysis. Unfortunately, excluding studies with missing SDs reduces the overall sample size (i.e. number of included studies) and can bias results (Kambach et al., 2020).

An alternative to excluding studies with incomplete data is to impute missing SDs via multiple imputation (MI; Ellington et al., 2015; Kambach et al., 2020). As a tool to handle missing data, MI was introduced to ecologists more than a decade ago (Nakagawa & Freckleton, 2008). However, MI is not widely used in the context of meta‐analysis likely for two major reasons. First, the implementation of MI is tedious because it involves three steps: (1) creating *m* (e.g. *m* = 100) versions of the dataset, each containing its own set of imputed values for the missing SDs, (2) analysing each of these *m* datasets separately, and (3) aggregating the *m* parameter estimates (e.g. regression coefficients) via Rubin's rules (Rubin, 1987) (for details, see Nakagawa, 2015; van Buuren, 2018). The second reason MI is not widely used in meta‐analysis is uncertainty around its implementation. For example, it is unclear if Rubin's rules are always appropriate for aggregating estimates of variance/heterogeneity (e.g. *τ* ^2^, *I* ^2^ and *R* ^2^) or information criteria (e.g. AIC, BIC; cf. Nakagawa & Freckleton, 2011). Furthermore, MI cannot easily be implemented for multilevel (mixed‐effects/hierarchical) meta‐analyses, and those implementations that do exist are limited to relatively simple models (van Buuren, 2018). For example, as far as we are aware, there is no off‐the‐shelf implementation of MI for the phylogenetic multilevel meta‐analytic models that are recommended for multi‐species meta‐analyses—a near universal feature of ecological meta‐analyses (Cinar et al., 2022).

Another alternative to excluding studies with missing SDs (i.e. complete‐case analysis) is to perform an ‘unweighted’ meta‐analysis with lnRR (Koricheva & Gurevitch, 2014; O'Dea et al., 2021). This approach does not include the sampling variances of effect sizes and thus does not require SDs. However, unweighted analyses are generally inferior to ‘weighted’ meta‐analyses for two reasons (cf. Buck et al., 2022). First, weighted meta‐analyses appropriately give more weight to the more precisely estimated effect sizes in the dataset (e.g. those studies with larger sample sizes and hence smaller sampling variances). This weighting improves precision of model parameter estimates, and imparts resilience to publication bias (Gurevitch et al., 2018; Hedges & Olkin, 1985), because smaller studies, which are down‐weighted in a weighted analysis, tend to be most affected by this phenomenon. This is an important consideration since publication bias is a common problem in ecology (e.g. Yang et al., 2022). Second, a weighted meta‐analytic model can also quantify heterogeneity (i.e. variation among effect sizes not due to sampling variance) while unweighted models cannot. Quantifying heterogeneity is essential because the overall mean effect size can only be appropriately interpreted in the context of the level of heterogeneity (Gurevitch et al., 2018; Hedges & Olkin, 1985; Nakagawa et al., 2017; Spake et al., 2022).

**FIGURE 1:**
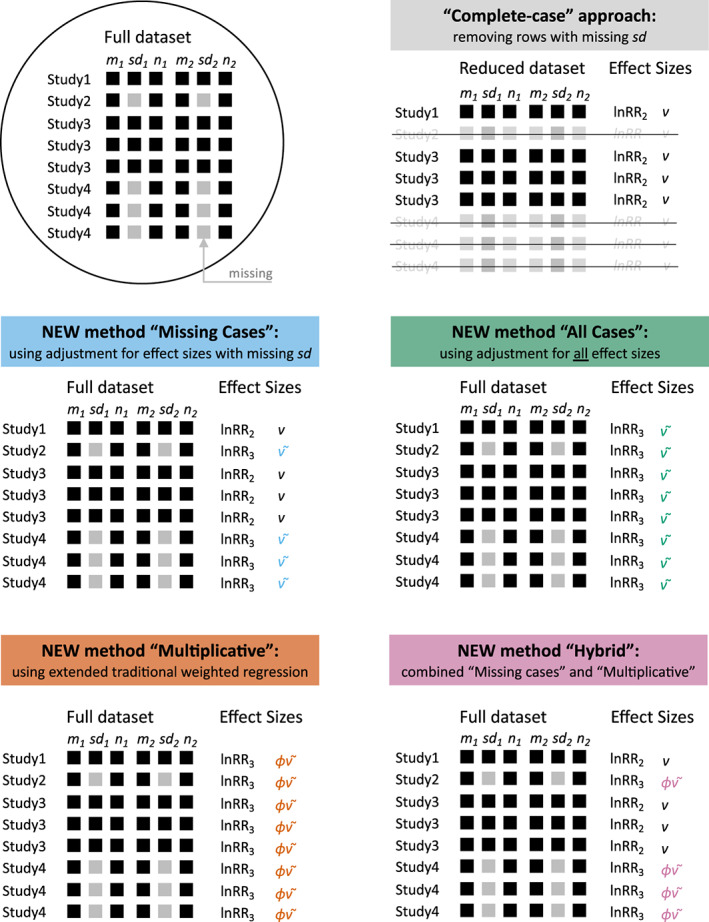
Visual schematics of a hypothetical dataset with missing standard deviations (SDs) and five different approaches used in this study, including 3 new methods. The symbols: lnRR_2_ (Equation 4), lnRR_3_ (Equation 6), v (Equation 5), $\tilde{v}$ (Equation 7), and $\phi \tilde{v}$ (Equation 12). Note that, under some circumstances, we could replace Equations 4 and 6 with Equation 1 while Equation 7 can be replaced by Equation 15 (see the text for more details).

Here, we propose four new methods for handling studies with missing SDs when the lnRR is the effect size of choice (Kambach et al., 2020; Koricheva & Gurevitch, 2014; Nakagawa & Santos, 2012). We note here that our methods do not readily extend to the SMD because the point estimate of SMD is extremely sensitive to the SD, which adds complexity. However, our methods integrate with formal meta‐analytic models, including traditional random‐effects models and the multilevel models that are often more appropriate in ecology (see Figure 1). We start with an adjusted sampling variance formula for lnRR developed by Doncaster and Spake (2018), which we improve and extend to provide two methods for handling missing SDs: using this adjustment only for effect sizes with missing SDs (the ‘missing‐cases’ method) and using this adjustment for all effect sizes regardless of missingness (the ‘all‐cases’ method). We then describe a third method that extends traditional weighted regression (the ‘multiplicative’ method). Finally, we combine the missing‐cases and multiplicative methods, to give a ‘hybrid’ method. To compare the performance of these four methods, we carried out a simulation study including a standard meta‐analytic model without missing SDs as a reference. Under a very broad range of simulated conditions, the all‐cases method performs best. Surprisingly, even with missing SDs, the all‐cases method outperforms the reference method with complete data. Finally, we make recommendations for future meta‐analyses. Importantly, we implement and illustrate these new methods via the widely used R package, *metafor* (Viechtbauer, 2010; all relevant data and code are available at a GitHub repository; see below).

## NEW STATISTICAL METHODS

### More precise sampling variances: The missing‐cases and all‐cases methods

The effect size statistic, lnRR, was first proposed by Hedges et al. (1999) as follows:

(1) $$\ln{\mathrm{RR}}_{1}=\ln\left(\frac{m_{1}}{m_{2}}\right),$$

(2) $$v\left(\text{lnRR}\right)=\frac{sd_{1}^{2}}{n_{1}m_{1}^{2}}+\frac{sd_{2}^{2}}{n_{2}m_{2}^{2}}=\frac{{\mathrm{CV}}_{1}^{2}}{n_{1}}+\frac{{\mathrm{CV}}_{2}^{2}}{n_{2}},$$

where *m*_1_ and *m*_2_ are the means of groups 1 and 2, respectively (e.g. experimental and control groups), *v* represents the sampling variance, *sd* and *n* are the corresponding SDs and sample sizes, respectively, and CV (*sd*/*m*) is the coefficient of variation.

However, when the sample size (*n*; i.e. number of replicates) per effect size is small, the CVs in Equation 2 are often imprecise. This is because the CV is based on *sd* and *m*, which are themselves estimates that become less precise with small sample sizes. If we assume the CV values for group 1 and group 2 are reasonably homogeneous across effect sizes (studies), we can obtain a single more precise estimate of CV^2^ by averaging across all values in the dataset (Doncaster & Spake, 2018; see also Hedges & Olkin, 1985; Hunter & Schmidt, 1990; Berkey et al., 1995):

(3) $$v^{*}\left(\text{lnRR}\right)=\frac{\sum_{i=1}^{K}\left({\mathrm{CV}}_{1i}^{2}\right)/K}{n_{1}}+\frac{\sum_{i=1}^{K}\left({\mathrm{CV}}_{2i}^{2}\right)/K}{n_{2}},$$

where ${\mathrm{CV}}_{1i}^{2}$ and ${\mathrm{CV}}_{2i}^{2}$ are the CVs from the *i*th study (study; *i* = 1, 2, …, *K*; we assume the number of effect sizes = the number of studies = *K*). Indeed, Doncaster and Spake (2018) have demonstrated that the use of Equation 3 over Equation 2 improves the accuracy and precision of the overall (meta‐analytic) mean estimate, especially when *n* is small (e.g. *n* = 3–10 observations, with *n*_1_ + *n*_2_ = 6–20). Notably, they also suggested this formula could be used when SDs are missing from some studies, although this application was not investigated by simulation.

Here, we propose two improvements to Equation 3. Using simulations, Lajeunesse (2015) showed that Equations 1 and 2 are biased when sample sizes are small to moderate, and that the following estimators—based on the second‐order Taylor expansion—can reduce these biases (see also Senior et al., 2020):

(4) $$\ln{\mathrm{RR}}_{2}=\ln\left(\frac{m_{1}}{m_{2}}\right)+\frac{1}{2}\left(\frac{{\mathrm{CV}}_{1}^{2}}{n_{2}}-\frac{{\mathrm{CV}}_{2}^{2}}{n_{1}}\right),$$

(5) $$v\left(\text{lnRR}\right)=\frac{{\mathrm{CV}}_{1}^{2}}{n_{1}}+\frac{{\mathrm{CV}}_{2}^{2}}{n_{2}}+\frac{{\mathrm{CV}}_{1}^{4}}{2n_{1}^{2}}+\frac{{\mathrm{CV}}_{2}^{4}}{2n_{2}^{2}}.$$

Therefore, unifying Equations 3 and 5, and using the square of the weighted average CV (rather than average of CV^2^, which is more sensitive to the assumption of normality; see Section “The accuracy and limitation of lnRR”) gives the following new estimators for the effect size and sampling variance:

(6) $$\ln{\mathrm{RR}}_{3}=\ln\left(\frac{m_{1}}{m_{2}}\right)+\frac{1}{2}\left(\frac{{\left[\sum_{i=1}^{K}\left(n_{1i}{\mathrm{CV}}_{1i}\right)/\sum_{i=1}^{K}n_{1i}\right]}^{2}}{n_{1}}-\frac{{\left[\sum_{i=1}^{K}\left(n_{2i}{\mathrm{CV}}_{2i}\right)/\sum_{i=1}^{K}n_{2i}\right]}^{2}}{n_{2}}\right),$$

(7) v˜lnRR=∑i=1Kn1iCV1i/∑i=1Kn1i2n1+∑i=1Kn1iCV2i/∑i=1Kn2i2n2+∑i=1Kn1iCV1i/∑i=1Kn1i42n12+∑i=1Kn2iCV2i/∑i=1Kn2i42n22.

We can use Equations 6 and 7 to calculate effect sizes and sampling variances when SDs are missing by simply imputing the pooled CV from the subset of studies that do report SDs. We call this approach as the ‘missing‐cases’ method because we only apply Equations 6 and 7 to studies with missing SDs, while the standard approach of Equations 4 and 5 are applied to studies that report SDs (see Figure 1 and Table 1 where we have consolidated information about the different methods and their assumptions).

**TABLE 1:** Equations and assumptions for different methods, including the case with no missing data (see also Figure 1)

Method	Point estimate^a^	Sampling variance (SD not missing)	Sampling variance (SD missing)	Assumptions in relation to sampling variance
Reference (No missing data)	Equation 4	Equation 5	Not applicable	Equation 5 estimates sampling variance well (observed mean and SD values are reasonable estimates of true values)
Missing cases	Equations 4 and 6	Equation 5	Equation 7	When SD values are missing, Equation 7 can estimate sampling variance for these missing cases well
All cases	Equations 4 and 6	Equation 7	Equation 7	Equation 7 estimates sampling variance better than Equation 5 regardless of missing SD
Multiplicative	Equations 4 and 6	Equation 12	Equation 12	Equation 12 estimates sampling variance better than Equation 5 or 7 regardless of missing SD
Hybrid	Equations 4 and 6	Equation 5	Equation 12	When SD is missing, Equation 12 can estimate sampling variance for these missing cases well (better than Equation 7)

^a^ Applying both Equations 4 & 6 (the latter for observations/rows with missing SD) or applying only Equation 6 (even for all studies where SDs are not missing) would make little difference for (effect size) point estimates, unless effect sizes fulfil Equation 14.

Alternatively, one may use Equation 7 for all effect sizes/studies regardless of the missingness of SDs; we call this approach the ‘all‐cases’ method (Table 1). The key difference between the missing‐ and all‐cases methods is that the former assumes that Equation 5 (which bases sampling variances on the study‐specific CVs) provides the best estimate of a given effect size's sampling variance, reverting to Equation 7 in cases where SDs are not available. In contrast, the all‐cases method assumes that Equation 7 always gives more precise estimates of the sampling variance. Two issues to note are: (1) it is important to use the square of the weighted average CV (Equations 6 & 7) rather than a weighted average of CV^2^; CV^2^ is very sensitive to non‐normally distributed effect sizes (with large outlying CVs) which might be generated from count data (see Section “The accuracy and limitation of lnRR” below), and (2) when we have multiple effect sizes per study (most meta‐analytic datasets in ecology; Nakagawa & Santos, 2012), we need to first calculate a weighted average of CVs within studies before taking the weighted average of these cross‐study CVs. Alternatively, we could estimate the weighted average using a multilevel meta‐analysis of lnCV (Nakagawa et al., 2015; cf. Vachon et al., 2019).

### A weighted‐regression‐like approach: The multiplicative method

In the absence of SDs, it has been suggested that information on sample sizes, which are more commonly available, can be used to approximate the sampling variances for lnRR (or SMD), using the inverse of the following (e.g. Gurevitch & Hedges, 2001; Lajeunesse, 2013; Rosenberg et al., 1997):

(8) $$\tilde{n}=\frac{n_{1}n_{2}}{n_{1}+n_{2}}.$$

However, treating Equation 8 (originally proposed in Hedges & Olkin, 1985) as an estimate of the ‘exact’ sampling variance is erroneous because it ignores the other terms in Equations 2 & 5 (i.e. mean and SD) (see the review by Kambach et al., 2020). A more realistic assumption is to treat 1/$\tilde{n}$ as proportional to the sampling variance; indeed, Equation 2 reduces to the inverse of Equation 8 (i.e. 1/$\tilde{n}$) when we set both CVs to 1. Weighted regression models, commonly used to correct for heteroscedasticity, make this assumption of proportionality. Note that this differs from the classical random‐effects meta‐analytical model, which assumes that the exact sampling variances are known (and not just up to a proportionality constant). Many ecologists are likely to be familiar with weighted regression models that specify sample sizes as weights (Fletcher & Dixon, 2012).

The simplest random‐effects meta‐analytic model using lnRR can be written as follows:

(9) $${\text{lnRR}}_{i}=\beta_{0}+s_{i}+m_{i},$$

$$s_{i}∼N\left(0,\sigma_{s}^{2}\right),m_{i}∼N\left(0,v_{i}\right),$$

where $\beta_{0}$ is the overall/average effect (or meta‐analytic mean); *s*_i_ is the between‐study effect for the *i*th effect size, sampled from a normal distribution with a mean of zero and variance $\sigma_{s}^{2}$ (sometimes referred to as $\tau^{2}$), *m*_*i*_ is the sampling error for the *i*th effect size, which is also normally distributed with variance equal to the *i*th sampling variance (note that *i* = 1, 2, …, *K*, the number of effect sizes = the number of studies). As mentioned earlier, this model assumes that the sampling variance of lnRR is known (i.e. either Equation 2 or 5 = $v_{i}$ in Equation 9). The ratio between $\sigma_{s}^{2}$ and the total variance is often used to quantify heterogeneity (*I* ^2^):

(10) $$I^{2}=\frac{\sigma_{s}^{2}}{\sigma_{s}^{2}+\bar{v}},$$

where $\bar{v}$ is known as the ‘typical’ (or ‘average’) sampling variance (originally referred to as ‘typical within‐study variance’; sensu Higgins & Thompson, 2002), which can be estimated in several ways (Xiong et al., 2010).

Unlike the meta‐analytic model above, in a weighted regression, the following is assumed:

(11) $$v_{i}=\phi \left(\frac{1}{{\tilde{n}}_{i}}\right),$$

where ϕ , which is estimated by the model, functions as a ‘multiplicative’ parameter fulfilling the assumption of proportionality (i.e. $1/{\tilde{n}}_{i}$ ∝ $v_{i}$ ). The key point here is that the missing‐ and all‐cases methods both assume that Equations 5 and/or 7 provide an accurate estimate of a study's sampling variance (Table 1). However, Doncaster and Spake's simulation suggests that the sampling variance (using Equation 3) is likely to be imprecise when sample sizes are small (e.g. *n*_1_ + *n*_2_ = 6–20). Therefore, it may instead be advisable to assume that $v_{i}^{*}$ (Equation 3) is proportional to the true sampling variance. In the case that we have missing data, we can extend the assumption of proportionality to Equation 7 to estimate the sampling variance as:

(12) $$v_{i}=\phi {\tilde{v}}_{i}.$$

Practically, this can be implemented as a version of a weighted‐regression model that estimates ϕ and assumes proportionality for the sampling variance as in Equation 12 (Figure 1). We refer to this as the ‘multiplicative’ method. This method also assumes that Equation 12 provides the best estimate of sampling variance for all studies/effect sizes regardless of SD missingness (Table 1).

### Combining missing‐cases and the multiplicative method: The hybrid method

In the multiplicative method, Equation 12 is used regardless of whether SDs are missing or not. We can, however, combine the missing‐cases and multiplicative methods together into a ‘hybrid’ method (Figure 1). In this case, when SDs are available, we can use Equation 5 to obtain the sampling variance of lnRR (along with Equation 4 for the point estimate). When SDs are missing, we can use the multiplicative method (Equation 12, for the sampling variance and Equation 6 for the point estimate). The hybrid method assumes that Equation 5 gives the best estimate of the sampling variances like the missing‐case method, but that Equation 12 is an acceptable substitute when SDs are missing. We can write the hybrid method, using a multilevel meta‐analysis (including modelling multiple effect sizes per study) as follows:

(13) $${\text{lnRR}}_{\mathrm{ij}}=\beta_{0}+s_{i}+u_{\mathrm{ij}}+m_{\mathrm{ij}},$$

$$s_{i}∼N\left(0,\sigma_{s}^{2}\right),u_{\mathrm{ij}}∼N\left(0,\sigma_{u}^{2}\right),m_{\mathrm{ij}}∼N\left(0,V\right)$$

where *s*_*i*_ is the between‐study effect for the *i*th study (*i* = 1, 2, …, *K*), normally distributed with a mean of 0 and variance $\sigma_{s}^{2}$ (often referred to as *τ* ^2^), *u*_*ij*_ is the between‐effect‐size effect (or within‐study effect) for the *j*th effect size in the *i*th study, distributed with a mean of zero and variance $\sigma_{u}^{2}$ (*j* = 1, 2, …, *L*_*i*_, where *L*_*i*_ denotes the number of effect sizes within the *i*th study), **V** is a diagonal matrix with $v_{\mathrm{ij}}$ (Equation 5) when no SDs are missing and $\phi {\tilde{v}}_{\mathrm{ij}}$ (Equation 12) for cases of missing SD. For example, when we have five effect sizes in three studies, **V** would be:

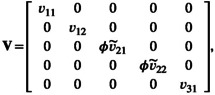

where 1st, 2nd and 5th effect sizes have SDs while the 3rd and 4th are without SDs, and as above, ϕ is estimated in the model. Because this model can account for non‐independence, it is appropriate in ecological meta‐analyses that include correlations among‐effect sizes such as when there is more than one effect size per study or species (Nakagawa & Santos, 2012; Noble et al., 2017; Nakagawa et al., 2022; but for a more complex model with **V** including covariances, or sampling variances with dependencies, see Appendix S1; https://alistairmcnairsenior.github.io/Miss_SD_Sim/). Importantly, all methods described in Table 1 can be used with multilevel meta‐analysis making this approach comparable with others.

## SIMULATION

### Simulation overview

We conducted a simulation study to compare the performance of the missing‐cases, all‐cases, multiplicative and hybrid methods on meta‐analytic datasets with varying proportions of missing SDs. A full description of the simulation is given in Appendix S2 (also see Table S1 for a summary of key parameters and their values). Briefly, meta‐analytic datasets were simulated with characteristics that are often seen in ecological studies. These characteristics included both high and low levels of among‐study heterogeneity in the overall mean, SD and sample size. We simulated datasets where the underlying studies typically had small (mean *n* = 5), and larger (mean *n* = 30) sample sizes. We implemented a version of the simulation where there were multiple effect sizes per study (i.e. non‐independence), which we refer to as Set I, and a version were there was just one effect size per study (i.e. complete independence), which we refer to as Set II.

For each simulated dataset, we analysed the full dataset using the conventional approach, before deleting SDs for 5%, 15%, 25% 35%, 45% or 55% of the studies. We treated 55% as the upper limit of missingness after consulting earlier surveys (e.g. Senior et al., 2016; Kambach et al., 2020; the latter found ecological meta‐analyses had missing SDs for up to 30% of cases). We then analysed each dataset using the ‘rma.mv’ function in *metafor* (Viechtbauer, 2010) with the four proposed methods for handling missing SDs. To evaluate performance, for each model, we calculated: (i) bias (as the difference between the estimated and the true, parametrised value) for the meta‐estimate of the overall mean effect size, (ii) bias for the (log) total amount of heterogeneity (*τ* ^2^ = $\sigma_{s}^{2}$ + $\sigma_{u}^{2}$ in Equation 13) and the estimated intra‐class correlation for study (ICC_*s*_), and (iii) coverage of 95% confidence intervals (CIs) for the overall mean.

### Simulation results

**FIGURE 2:**
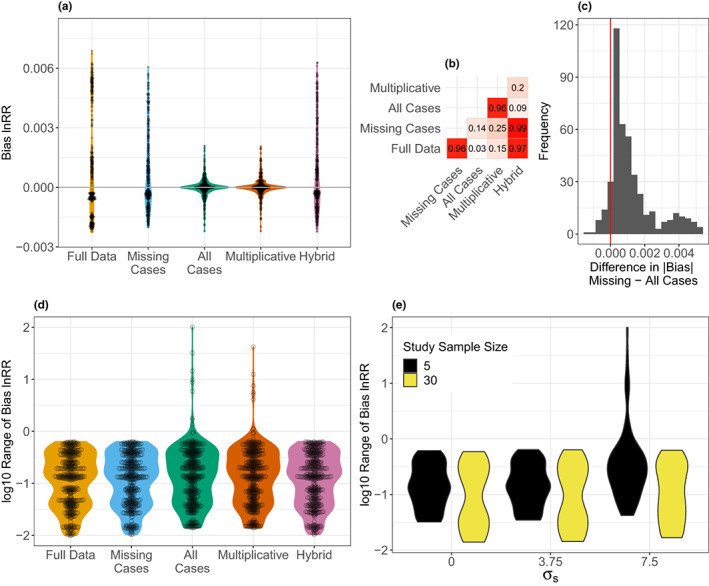
Results on overall meta‐analytic mean from multi‐level meta‐analytic models: (a) violin plot showing the distribution of median bias in the estimated effect under each simulated condition as a function of the method used to handle missing data (distribution assuming full data shown for reference). (b) Pairwise correlations between the degree of bias under each simulated condition for each method. (c) Distribution of the difference between the missing‐cases and all‐cases methods in the absolute degree of bias under each condition (positive values indicate greater median bias under the missing‐cases method). (d) Violin plot showing the distribution of range bias (log_10_ transformed) in the estimated effect under each simulated condition as a function of the method used to handle missing data. (e) Violin plot showing the distribution of range bias (log_10_ transformed) in the estimated effect using the all‐cases method under each simulated condition as a function of the degree of heterogeneity in SDs among studies under two different (within‐)study sample size conditions. Our plots were drawn using the R package ggplot2 (Wickham, 2009).

Figure 2a shows the distribution of median bias in estimated overall effects under each simulated condition with complete data and using the four different methods for missing SDs. Even with full data, upward and downward biases were possible for the estimated effect size, and this was also observed in the analyses using the missing‐cases and hybrid methods to handle missing SDs. Notably, even at its most extreme, this bias only amounted to a little over 2% of the true effect size and was usually ~0.5%, meaning all the proposed methods performed well (all methods had a median bias across conditions <0.0001). Nonetheless, the all‐cases and multiplicative methods, both of which use the weighted average CV to estimate the sampling variance for all effect sizes regardless of missingness, yielded the lowest bias on average and were less variable than other methods (Figure 2a). The all‐cases and multiplicative methods were consistently less biased than the other approaches, regardless of the degree of missingness (Figure S4a). The degree of bias across conditions in the full data analysis correlated strongly with that of bias from the missing‐cases and hybrid methods, while bias in the all‐cases and mulitplicative methods correlated strongly with each other (Figure 2b). This observation suggests that the methods fall into two classes that perform similarly across situations: the all‐cases and multiplicative methods and the missing‐cases and hybrid methods. Contrasting the missing‐cases and all‐cases methods, the absolute level of bias in the missing‐cases method was almost always higher than that for the all‐cases method (Figure 2c). Further, where the all‐cases method had a higher bias than the missing‐cases method, this difference was small (Figure 2c). Although the all‐cases and multiplicative methods outperformed the other approaches on average, they yielded extremely biased estimates on rare occasions; Figure 2d shows the range in bias among the individual replicates under each simulated condition as a function of the different methods. With the all‐cases method, large ranges in bias only occurred when the SDs among studies were highly heterogeneous, and within‐study sample sizes were low (Figure 2e).

**FIGURE 3:**
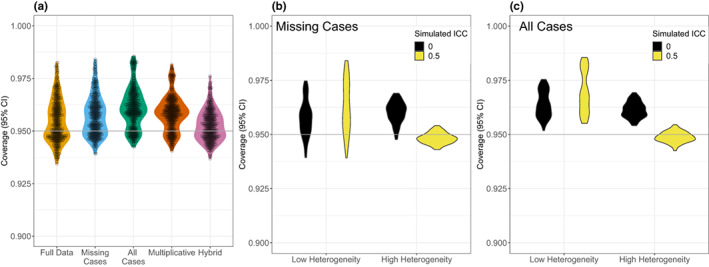
Results on coverage from multi‐level meta‐analytic models: (a) Violin plot showing the distribution of coverage of 95% CIs under each simulated condition as a function of the method used to handle missing data (distribution assuming full data shown for reference). (b) Violin plot showing the distribution of coverage under each simulated condition as a function of the simulated level of total heterogeneity and the ICC for study using the missing‐cases method to handle missing SDs. (c) Violin plot showing the distribution of coverage under each simulated condition as a function of the simulated level of total heterogeneity and the ICC for study using the all‐cases method to handle missing SDs. In (b) and (c), low heterogeneity is *τ* ^2^ = 9 × 10^−6^ (or *τ* / *θ* = 0.01), and high heterogeneity is *τ* ^2^ = 0.09 (or *τ*/*θ* = 1).

All methods for handling missing data, and the full data analyses, could produce 95% CIs that were too narrow, or wide under different scenarios (Figure 3a). The full data, and the missing‐cases and hybrid methods tended to produce CIs that were too narrow, whereas the all‐cases and multiplicative methods were prone to producing wider CIs (Figure 3a and Figure S4b). Again, contrasting the missing‐cases and all‐cases method, the all‐cases method tended to produce CIs that were too wide when the heterogeneity among studies is low (Figure 3b,c). However, where total heterogeneity is high, the all‐cases method performs as well as the missing‐cases method (Figure 3b,c).

**FIGURE 4:**
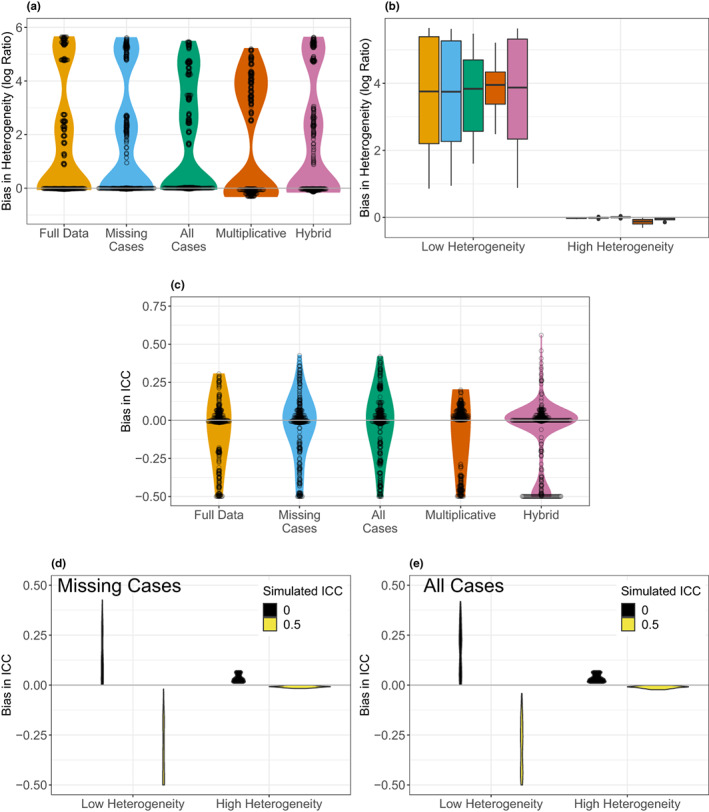
Results on heterogeneity from multi‐level meta‐analytic models: (a) violin plot showing the distribution of median bias in the estimated heterogeneity under each simulated condition as a function of the method used to handle missing data (distribution assuming full data shown for reference). Bias in heterogeneity is calculated as the log ratio of the estimated and parametrised value. (b) Box plot showing the median bias in estimated heterogeneity under each simulated condition as a function of the method used to handle missing data (colours as in panel a), and the simulated level of heterogeneity. (c) Violin plot showing the distribution of the median bias in the estimated ICC for study under each simulated condition as a function of the method used to handle missing data. Bias in the ICC was calculated as the difference between the estimated and parameterised value. (d) Violin plot showing the distribution of the median bias in the estimated ICC for study under each simulated condition as a function of the simulated level of total heterogeneity and the ICC for study using the missing‐cases method to handle missing SDs. (e) Violin plot showing the distribution of the median bias in the estimated ICC for study under each simulated condition as a function of the simulated level of total heterogeneity and the ICC for study using the all‐cases method to handle missing SDs. In (d) and (e), low heterogeneity is *τ* ^2^ = 9 × 10^−6^ (or *τ* / *θ* = 0.01), and high heterogeneity is *τ* ^2^ = 0.09 (or *τ*/*θ* = 1).

Figure 4a shows the median bias in the estimated heterogeneity under each condition and method. Under most conditions, the missing‐cases, all‐cases and hybrid methods estimated heterogeneities with little bias, but could also overestimate the total heterogeneity, although to a similar degree to the full data analysis (Figure 4a). The multiplicative method tended to underestimate heterogeneity (Figure 4a). Any bias in the estimation of heterogeneity was independent of the actual level of missingness (Figure S4c). Overestimation of heterogeneity occurred where the actual level of heterogeneity was low (Figure 4b). On average, most methods did a good job of partitioning heterogeneity between the within‐ and among‐study levels, although the multiplicative method displayed a slight bias (Figure 4c). Under some circumstances, all methods could be biased in partitioning heterogeneity (Figure 4c). As an example, the missing‐cases and all‐cases methods were prone to biased partitioning when the total heterogeneity was low; overestimating the ICC when the simulated study effect was absent and underestimating when it was present (Figure 4d,e).

In summary, although the all‐cases method performed with the least bias under the broad range of simulated conditions tested, all the methods fared surprisingly well, compared with the full data analysis (see Discussion for more). The results presented here pertain to the performance of these methods in the context of multilevel meta‐analytic models (Equation 13, which models non‐independence). However, these conclusions are mirrored for traditional random‐effects models (i.e. analyses without non‐independence; Figures 2, 3, 4 vs Figures S1–S3).

## IMPLEMENTATION

### The accuracy and limitation of lnRR

The accuracy of the sampling variance for lnRR depends on whether lnRR is normally distributed. Hedges et al. (1999) suggested a simple test to check the assumption of normality based on Geary (1930), who originally advocated screening for effect sizes with $\sqrt{n}/\mathrm{CV}\geq 3$ . This test was improved by Lajeunesse (2015) as:

(14) $$\frac{1}{\mathrm{CV}}\left(\frac{4n^{\frac{3}{2}}}{1+4n}\right)\geq 3.$$

If many effect sizes fail to fulfil this relationship, then, meta‐analytic results are unlikely to be robust. Lajeunesse (2015) suggests a sensitivity analysis, which excludes effect sizes that fail Equation 14. However, such tests are rarely used. Count data and related types (e.g. counts per a given time and space), which are extremely common in ecology (Spake et al., 2021), may often fail Equation 14. This is because such data is usually over‐dispersed, meaning CV >1. For example, it is not uncommon for count data to have CV = 5, especially when the mean is close to zero (cf. Lajeunesse, 2015). When CV = 5, the sample sizes need to be >226 for each group to pass Equation 14, which would be difficult for most ecological studies to attain.

All meta‐analyses of lnRR are sensitive to the assumption of normality to some degree, but our proposed formulations may be more sensitive because the Taylor expansion used in Equations (4), (5), (6), (7) assumes normality. Therefore, it may be advisable to use Equation 1 for the point estimate and the following estimator of the sampling variance (rather than Equation 7) when many effect sizes fail Geary's test (see also Table S2):

(15) $$\tilde{v}\left(\text{lnRR}\right)=\frac{{\left[\sum_{i=1}^{K}\left(n_{1i}{\mathrm{CV}}_{1i}\right)/\sum_{i=1}^{K}n_{1i}\right]}^{2}}{n_{1}}+\frac{{\left[\sum_{i=1}^{K}\left(n_{1i}{\mathrm{CV}}_{2i}\right)/\sum_{i=1}^{K}n_{2i}\right]}^{2}}{n_{2}}.$$

This formula still relies on the first‐order Taylor expansion, but not the second‐order, and is therefore less sensitive than Equation 7 to violations of Geary's test. Other limitations (and advantages) of lnRR are discussed elsewhere (e.g. Spake et al., 2021; Yang et al., 2022).

### Worked examples

Bird et al. (2019) conducted a meta‐analysis exploring the impacts of competition on herbivorous insect fitness when occupying a host plant with another species or in isolation. In brief, they collected data on a series of fitness measurements (e.g. abundance, body size, development time, fecundity; see Table 2 in Bird et al., 2019) and quantified the impact of competition on those measures using phylogenetic multilevel meta‐analyses (Cinar et al., 2022; Appendix S1).

**TABLE 2:** Results from the re‐analyses of a subset of data from Bird et al. (2019) using the methods we propose to deal with missing SD data estimating the overall effects of competition on focal insect abundance (LCI = lower, or 2.5%, confidence limit; UCI = upper, or 97.5%, confidence limit)

Method	Est.	SE	95% LCI	95% UCI
Full data	0.202	0.085	0.036	0.369
Complete case	0.176	0.102	−0.024	0.377
Missing cases	0.186	0.091	0.008	0.364
All‐cases	0.146	0.096	−0.043	0.334
Multiplicative	0.192	0.083	0.03	0.354
Hybrid	0.185	0.086	0.017	0.353

For demonstration purposes, we focused on the largest dataset that used measures of abundance (population size). We restricted our analysis to data on the ratio scale (i.e. having true zero, which is a condition required for lnRR) and those effect sizes that passed the ‘improved’ Geary's test (Equation 14 above), giving a total of 173 effect sizes from 62 studies. We use a multilevel meta‐analytic model (Equation 13) to estimate the overall impact of competition on focal insect fitness (i.e. intercept or overall meta‐analytic mean) while controlling for phylogeny, research group and research year (as per the analysis by Bird et al., 2019). We then introduced missing data at the study (article) level, so that a randomly selected ~20% of articles had effect sizes with missing SD in the control and experimental groups; a scenario that is typical of many meta‐analyses (cf. Kambach et al., 2020).

An analysis of these data applying the different methods compared to the full data is provided in Table 2. We can see that the complete‐case analysis (excluding all data with missing SDs) gives slightly larger confidence intervals that cross zero, and a reduction in the meta‐analytic mean effect size, relative to most of the other methods. The missing‐cases, multiplicative and hybrid methods all suggest the overall meta‐analytic is slightly larger and result in greater precision around this estimated effect size than the complete‐case analysis. The all‐cases method had the smallest overall effect size magnitude, which was not significantly different from zero, while the other three methods yielded mean estimates that were significant (see Discussion). Using this example, we show how each approach is implemented in the supplement (Appendix S3) along with an additional example (McDonald et al., 2019; Appendix S4).

## DISCUSSION

In this study, we developed new methodological procedures to handle missing SDs in meta‐analyses of lnRR. Our methods will enable researchers, including ecologists and evolutionary biologists alike, to incorporate studies with missing SDs in their meta‐analyses, while also using appropriately weighted formal meta‐analyses rather than unweighted counterparts. Our simulation suggested that the least biased estimates were obtained by the ‘all‐cases’ method. This method uses the weighted average CV (estimated from those studies with SDs) to calculate point estimates and sampling variances for all effect sizes, regardless of missingness in SD (Table 1). In terms of implementation, this is also the easiest method of those that we describe (see Appendixes S3–S4).

The all‐cases method effectively uses ‘single imputation’ (rather than ‘multiple imputation’), and single imputations are generally believed to fare worse than meta‐analysis with full data (using Equations 4 & 6, see Table 1; Nakagawa & Freckleton, 2008; Nakagawa, 2015; van Buuren, 2018; Kambach et al., 2020; see also Fletcher & Dixon, 2012). Yet, this is not what we found. In their previous simulation, Doncaster and Spake (2018) found that Equation 3, which uses the average CV for all effect sizes, performed better than analysis with Equation 2, which uses study‐specific CVs. Thus, on reflection, we might have expected the all‐cases method to do well (see also Lin & Aloe, 2021).

The all‐cases method and Doncaster and Spake's procedure (i.e. using Equation 3 rather than Equation 2) perform well because, even where they are reported, the CV values from individual studies are often imprecise due to the small within‐study sample size. This, in turn, results in imprecise estimates of the sampling variance. However, using a pooled CV improves estimates of the sampling variance, with benefits to the downstream analyses. Of relevance, another simulation study by Bakbergenuly et al. (2020) suggests that sample size (more precisely, $\tilde{n}$ as in Equation 8) is the most important component of weighting in the analysis of lnRR. This insight explains why the all‐cases and multiplicative methods do well even in simulations that violate the assumption that CV is homogenous across studies, especially when the number of effect (*K*) is large (see more for this point below).

It is important to note that our simulation built on those in Doncaster and Spake (2018) in at least three respects. First, Doncaster and Spake (2018) never tested how their method fared with missing data. Second, our simulation uses multilevel models that are now being applied to many ecological datasets. Third, our simulation has shown that, as well as reducing bias in overall estimates, using a pooled CV does not compromise the accuracy of heterogeneity estimates (i.e. variance components). Between our work and the previous publication by Doncaster and Spake (2018), we have established that using a cross‐study averaged CV in the estimation of effect sizes can improve ecological meta‐analyses in a range of realistic scenarios.

Incidentally, Doncaster and Spake (2018) are not the first to use the ‘averaging’ method. For example, Hedges and Olkin (1985) also proposed to use the average of the observed standardised mean differences in the computation of their sampling variances when meta‐analysing a large number of small studies. Also, Hunter and Schmidt (1990) proposed to use the weighted average of correlations in the sampling variance for the correlation coefficient. Similarly, Berkey et al. (1995) showed that using averages of counts or proportions in the Equations for computing the sampling variances of log relative risks and odds ratios led to less biased estimates.

There were two conditions where the all‐cases method could result in biased estimates. The first scenario is when CVs are very different between studies, and within‐study sample size is relatively small. As discussed below, parallel analysis with the missing‐cases method (or alternatively the hybrid method, although the latter is more difficult to implement) could help establish the stability of meta‐analytic results. In addition, a meta‐analysis of lnCVR (log CV ratio) or lnCV (log CV) could help to evaluate how large the between‐study variance in CV is (Nakagawa et al., 2015; Senior et al., 2020). Large variation in between‐study CVs would violate our assumption that the CV is relatively constant (cf. Nakagawa et al., 2015). Note, however, that our simulation shows this assumption is less important when studies have larger sample sizes. The second scenario is when there is very low total heterogeneity (*τ* ^2^ = $\sigma_{s}^{2}$ + $\sigma_{u}^{2}$, which usually translates to low *I* ^2^; see Higgins et al., 2003; Nakagawa & Santos, 2012; also see Borenstein et al., 2017). As mentioned earlier, heterogeneity is typically high in meta‐analyses in ecology (and evolutionary biology). Indeed, Senior et al. (2016) showed that on average, ecological and evolutionary meta‐analyses have high heterogeneity with *I* ^2^ of around 90%. Therefore, the second scenario may not be of concern to most ecologists.

Based on the simulation results alone it would be natural to recommend the use of the all‐cases method as the default. While we believe the all‐cases method is generally the most robust, we advocate that analysts take caution and adopt the following procedure: One should conduct a meta‐analysis using both the missing‐cases and all‐cases methods in tandem, which is very straightforward (see Supplementary Information). If the results of the two methods are qualitatively the same (e.g. both statistically significant, with similar effect size magnitudes), one can present the all‐cases method in confidence. If, however, the results are qualitatively different, both results should be presented (e.g. our worked example: see Table 2). In such a case, one should conclude carefully and emphasise uncertainty about their results. An analysis of the heterogeneity among CVs may help guide the user to decide which results to favour; if the CVs are quite different across studies, results from the missing‐cases method may be more reliable (see above).

Notably, our simulation assumes that SDs are missing completely at random. Therefore, when cases with missing SDs are non‐random and have consistently higher or lower CVs than cases with SDs, one could use the hybrid method. The hybrid method was shown to work as well as the all‐cases method, but this method also can adjust for higher or lower CVs via the multiplicative term ϕ (see Equations 12). A complication here is that one is unlikely to ever know what the CVs of missing cases are, and therefore may have to just try the hybrid method to find out (i.e.,ϕ being more or less than 1). We do however re‐emphasise that all the methods we proposed work well under many conditions (i.e., were not more/less biased than an analysis of the full data). Regardless, it is important to report the % of missing SDs, and which methods have been used to handle missing data, in accordance with the PRISMA‐EcoEvo (Preferred Reporting Items for Systematic reviews and Meta‐Analyses in Ecology and Evolutionary biology) reporting guidelines (O'Dea et al., 2021).

Finally, our proposed methods are easy to implement and readily extend to a host of complex models. We hope that meta‐analysts in ecology and evolution will adopt these two new approaches to improve their meta‐analytic estimation, especially the all‐cases approach which performs well even in the absence of missing data. Importantly, we should also all be aware of the limitations of the lnRR for meta‐analyses, for example, by more routinely evaluating the underlying assumptions using the improved Geary's test.

## AUTHOR CONTRIBUTIONS

SN and WV came up with the initial idea and statistical methods, which were discussed and expanded by the other co‐authors. AMS led the simulation study, and DWAN put together Supplementary Information with the others' inputs. SN, AMS & DWAN wrote the first draft and all the authors edited and commented on earlier versions of the manuscript.

## CONFLICT OF INTEREST

We declare no conflict of interest.

### PEER REVIEW

The peer review history for this article is available at https://publons.com/publon/10.1111/ele.14144.

### OPEN RESEARCH BADGES

This article has earned Open Data and Open Materials badges. Data and materials are available at: 10.5281/zenodo.7302038 and https://osf.io/h9x6w/.

## Supporting information


Supporting information S1.

## Data Availability

All relevant code and data can be found at Zenodo (DOI: 10.5281/zenodo.7302038).
