# Supplementary material for: A robust and readily implementable method for the meta‐analysis of response ratios with and without missing standard deviations
Source: Ecol Lett. 2022 Dec 26;26(2):232–44. doi: 10.1111/ele.14144 (PMC10108319; doi:10.1111/ele.14144)
Supplement: Supplementary file 1 — Supporting information S1. [file ELE-26-232-s001.docx]

**Supplementary tables and figures**

**A robust and readily implementable method for the meta-analysis of response ratios with and without missing standard deviations**

Shinichi Nakagawa, Daniel W. A. Noble, Malgorzata Lagisz, Rebecca Spake, Wolfgang Viechtbauer & Alistair M. Senior

**Table S1.** Variables/parameters in simulations.

| **Variable (Notation)** | **Description and details** | **Value(s)** |
| --- | --- | --- |
| % Studies Missing SD | Percentage of studies that have missing SDs | 5, 15, 25, 35, 45 or 55 |
| Overall Effect Size (*θ*) | The overall mean lnRR effect size | 0.3 |
| Number of Studies (*K*) | Total number of studies within the meta-analytic dataset | 12, 30, 100 |
| Standard Deviation in Study (*S*) | The within-study SDs. Individual within-study SDs were randomly distributed following a Gamma distribution | Random with a mean (*μ_S_*) of 15 and a SD of either 10^-10^, 3.75 or 7.5 |

Table S2 Recommendations for the use of equations when observations (effect sizes) fail Geary’s test; note $\phi$ is a multiplicative factor as in Equation 12 (cf. Table 1).

| **Method** | **Point estimate** | **Sampling variance**  **(SD not missing)** | **Sampling variance**  **(SD missing)** |
| --- | --- | --- | --- |
| Missing cases | Equation 1 | Equation 2 | Equation 15 |
| All cases | Equation 1 | Equation 15 | Equation 15 |
| Multiplicative | Equation 1 | Equation 15 x $\phi$ | Equation 15 x $\phi$ |
| Hybrid | Equation 1 | Equation 2 | Equation 15 x $\phi$ |

**Figure S1**

Results on overall meta-analytic mean from random-effects meta-analytic models: A) Violin plot showing the distribution of median bias in the estimated effect under each simulated condition as a function of the method used to handle missing data (distribution assuming full data shown for reference). B) Pairwise correlations between the degree of bias under each simulated condition for each method. C) Distribution of the difference between the missing-cases and all-cases methods in the absolute degree of bias under each condition (positive values indicate greater median bias under the missing-case methods). D) Violin plot showing the distribution of range bias (log_10_ transformed) in the estimated effect under each simulated condition as a function of the method used to handle missing data. E. Violin plot showing the distribution of range bias (log_10_ transformed) in the estimated effect under each simulated condition using the all-cases method to handles missing SDs as a function of the degree of heterogeneity in SDs among studies under two different (within-)study sample size conditions.

**Figure S2**

Results on coverage from random-effects meta-analytic models: A) Violin plot showing the distribution of coverage of 95% CIs under each simulated condition as a function of the method used to handle missing data (distribution assuming full data shown for reference). B) Violin plot showing the distribution of coverage under each simulated condition as a function of the simulated level of total heterogeneity and the ICC for study using the missing-case method to handle missing SDs. C) Violin plot showing the distribution of coverage under each simulated condition as a function of the simulated level of total heterogeneity and the ICC for study using the all-cases method to handle missing SDs. In B and C, low heterogeneity is *τ*^2^ = 9×10^-6^ (or *τ* / *θ* = 0.01), and high heterogeneity is *τ*^2^ = 0.09 (or *τ* / *θ* = 1).

**Figure S3**

Results on coverage from random-effects meta-analytic models: A) Violin plot showing the distribution of median bias in the estimated heterogeneity under each simulated condition as a function of the method used to handle missing data (distribution assuming full data shown for reference). Bias in heterogeneity is calculated as the log ratio of the estimated and parametrized value. B. Box plot showing the median bias in estimated heterogeneity under each simulated condition as a function of the method used to handle missing data (colours as in panel A), and the simulated level of heterogeneity.

**Figure S4**

Bias in A) overall meta-analytic mean estimation, B) coverage and C) heterogeneity, as function of the method used to handle missing SDs and the percentage of studies with missing SDs in the simulated dataset. Note that for the full data analysis no studies have missing SDs and thus no trend is expected. Random ‘jitter’ has been added to the *x*-axis to make overlaying points visible. Fitted lines are based on a generalised additive model (GAM) implemented using the ‘geom_smooth’ function in ggplot2.
